# LONG-DISTANCE CAREGIVING: MENTAL HEALTH CONSEQUENCES AND USE OF RESOURCES

**DOI:** 10.1093/geroni/igz038.2055

**Published:** 2019-11-08

**Authors:** Verena R Cimarolli, Amy Horowitz, Rachel Pruchno

**Affiliations:** 1 LeadingAge LTSS Center @UMass Boston, Washington, District of Columbia, United States; 2 Fordham University, New York, New York, United States; 3 Rowan University, Stratford, New Jersey, United States

## Abstract

Long-distance caregiving (LDC) is a growing phenomenon and common experience for caregivers of frail older adults. In fact, 11% of family caregivers in the US live more than two hours distance from the care recipient (CR). Unfortunately, there is a paucity of research on unique experiences of LDCs and the impact of LDC on the mental health of LDCs. This symposium presents findings from the NIA funded Fordham Long-Distance Caregiving Study (R21AG050018) analyzing data of 304 long-distance caregivers (LDCs). The overall study goal was to better understand how LDCs deal with the structural constraint of distance, and to examine LDC consequences and resources. First, Horowitz presents the study background, characteristics of the sample, and provides a description of the unique experiences of LDCs. Next, Cimarolli concentrates on the Sociocultural Stress Process Model applied to LDC. Her study tested the impact of LDC on mental health and investigated resources (e.g., coping skills) which could mediate the association between caregiving stressors and mental health outcomes. The third paper (Falzarano) presents data related to satisfaction with formal service providers for four subgroups of LDCs based on CR residence and dementia status. Finally, Jimenez focuses on the characteristics of LDCs’ network of other informal caregivers (IC) providing assistance to the CR and factors are associated with more help received from other ICs. Dr. Pruchno, an expert in caregiving research, will discuss study findings. The symposium provides insights into unique experiences of LDCs, the impact of LDC on mental health, and resource use among LDCs.

